# Crystal structure of 2,5-di­hydroxy­terephthalic acid from powder diffraction data

**DOI:** 10.1107/S2056989022009409

**Published:** 2022-09-30

**Authors:** Joshua D. Vegetabile, James A. Kaduk

**Affiliations:** a Department of Chemistry, North Central College, 131 S. Loomis, St., Naperville IL, 60540 , USA; University of Aberdeen, Scotland

**Keywords:** powder diffraction, 2,5-di­hydroxy­terephthalic acid, Rietveld refinement, density functional theory

## Abstract

The crystal structure of anhydrous 2,5-dhy­droxy­terephthalic acid, C_8_H_6_O_6_, was solved and refined using laboratory X-ray powder diffraction data, and optimized using density functional techniques. The published structure of 2,5-di­hydroxy­terephthalic acid dihydrate was also optimized. The carb­oxy­lic acid groups form strong hydrogen bonds, which form centrosymmetric rings with graph set *R* ^2^_2_(8). These hydrogen bonds link the mol­ecules into chains along [011]. There is an intra­molecular O—H⋯O hydrogen bond between the hydroxyl group and the carbonyl group of the carb­oxy­lic acid. The hydrogen bonding in the dihydrate is very different. Although the intra­molecular hy­droxy/carb­oxy­lic acid hydrogen bond is present, the water mol­ecule acts as an acceptor to the carb­oxy­lic acid and a donor to two other oxygen atoms. The carb­oxy­lic acid groups do not inter­act with each other directly.

## Abstract 2

The crystal structure of anhydrous 2,5-dhy­droxy­terephthalic acid, C_8_H_6_O_6_, was solved and refined using laboratory X-ray powder diffraction data, and optimized using density functional techniques.

## 1. Chemical context

2,5-Di­hydroxy­terephthalate (C_8_H_4_O_6_^2–^; dhtp) is of current inter­est as a linker in metal–organic frameworks (MOFs). It can add extra functionality to alter adsorption and catalytic properties. In an attempt to replicate the ionothermal preparation of the Co-dhtp MOF Co_2_(dobdc)-ST (Azbell *et al.*, 2022 ▸), an unexpected product was obtained, namely anhydrous 2,5-dhy­droxy­terephthalic acid, C_8_H_6_O_6_, (I). 
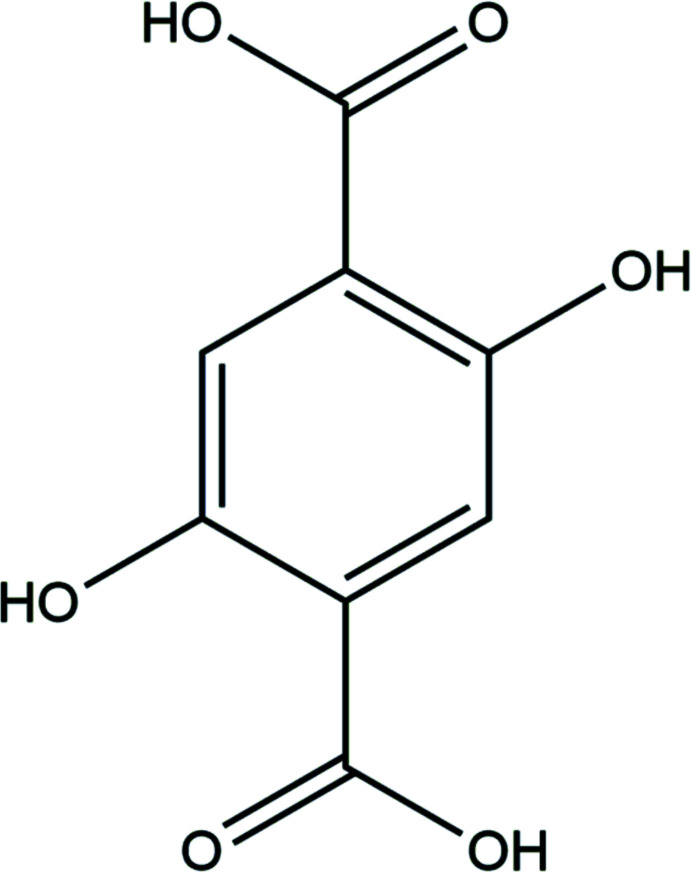


**Figure 1:**
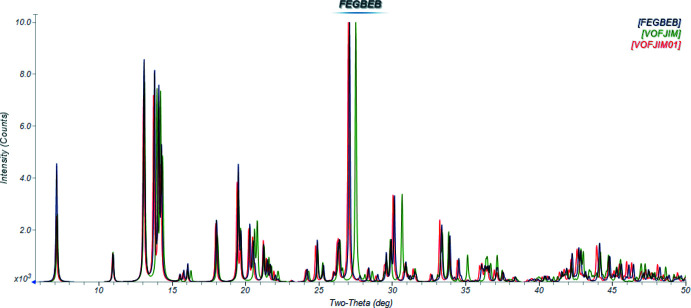
Calculated (using *Mercury;* Macrae *et al.*, 2020 ▸) powder diffraction patterns (Cu *K*α radiation) for CPO-27-Co [FEGBEB (Gen, 2017 ▸), VOFJIM (Rosnes *et al.*, 2019 ▸) and VOFJIM01 (Ayoub *et al.*, 2019 ▸)]. The differences in peak positions result from the different temperatures of the diffraction studies. Image generated using *JADE Pro* (MDI, 2022 ▸).

The crystal structures of three Co-dhtp MOFs have been reported: Cambridge Structural Database refcodes FEGBEB (Gen, 2017 ▸), VOFJIM (Rosnes *et al.*, 2019 ▸) and VOFJIM01 (Ayoub *et al.*, 2019 ▸). The calculated powder patterns of these three compounds, which have been given the name CPO-27-Co, indicate that they have the same structure (Fig. 1 ▸).

## 2. Structural commentary

**Figure 2:**
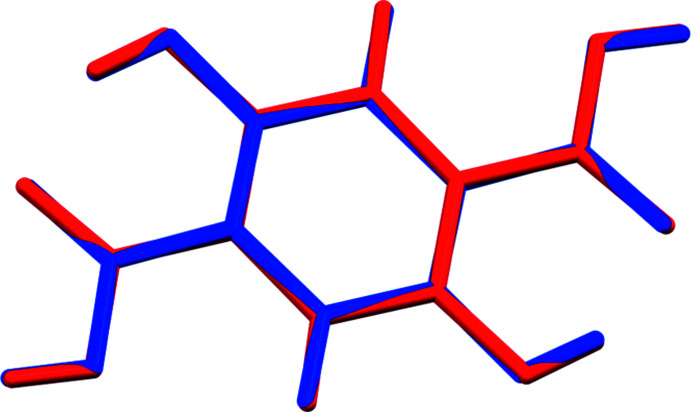
Comparison of the Rietveld-refined (red) and *VASP*-optimized (blue) structures of anhydrous 2,5-di­hydroxy­terephthalic acid. The r.m.s. Cartesian displacement is 0.053 Å. Image generated using *Mercury* (Macrae *et al.*, 2020 ▸).

**Figure 3:**
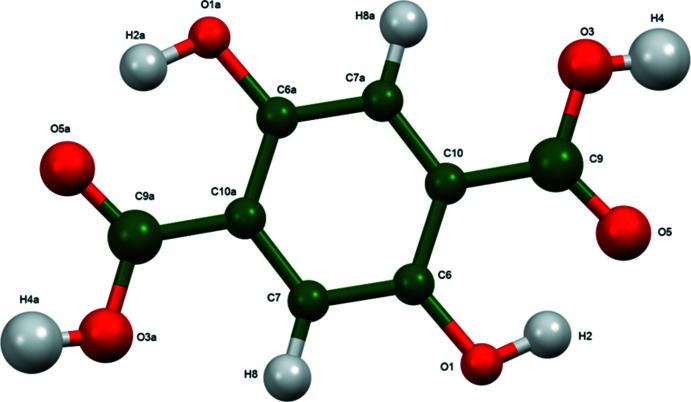
The full 2,5-di­hydroxy­terephthalic acid mol­ecule, with the atom numbering. The atoms are represented by 50% probability spheroids. Image generated using *Mercury* (Macrae *et al.*, 2020 ▸). Symmetry code: (*a*) 1 − *x*, 1 − *y*, 1 − *z*.

**Figure 4:**
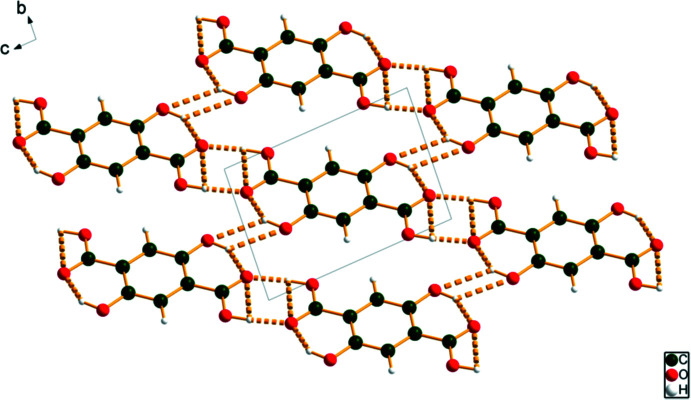
The crystal structure of anhydrous 2,5-di­hydroxy­terephthalic acid, viewed down the *a*-axis. Image generated using *DIAMOND* (Crystal Impact, 2022 ▸).

Compound (I) crystallizes in the triclinic space group *P* 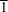 with half a mol­ecule in the asymmetric unit. The root-mean-square Cartesian displacements of the non-H atoms in the Rietveld-refined and *CRYSTAL17*-optimized structures is 0.053 Å (Fig. 2 ▸). The good agreement provides strong evidence that the structure is correct (van de Streek & Neumann, 2014 ▸). The *CRYSTAL17* and *VASP*-optimized structures are essentially identical (r.m.s. displacement = 0.031 Å). This discussion concentrates on the *CRYSTAL17*-optimized structure. The full mol­ecule (with atom numbering) is illustrated in Fig. 3 ▸ and a view of the packing down the *a*-axis direction is shown in Fig. 4 ▸.

All of the bond distances, angles, and torsion angles fall within the normal ranges indicated by a *Mercury* Mogul geometry check (Macrae *et al.*, 2020 ▸). The plane of the phenyl ring lies approximately on the (98 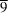 ) Miller plane. The peak profiles are dominated by anisotropic microstrain broadening: the average microstrain is 8362 ppm.

The Bravais–Friedel–Donnay–Harker (Bravais, 1866 ▸; Friedel, 1907 ▸; Donnay & Harker, 1937 ▸) morphology suggests that we might expect platy (with {001} as the major faces) morphology for this crystal. A 4th order spherical harmonics preferred orientation model was included in the refinement. The refined texture index was 1.059 (2), indicating that preferred orientation was small for this capillary specimen. In flat plate specimens examined in Bragg–Brentano geometry using Cu radiation, the preferred orientation tended to be higher.

## 3. Supra­molecular features

**Figure 5:**
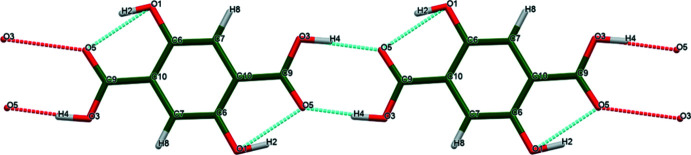
The hydrogen bonds in the structure of anhydrous 2,5-di­hydroxy­terephthalic acid. Image generated using *Mercury* (Macrae *et al.*, 2020 ▸).

In the extended structure of (I), the carb­oxy­lic acid groups form strong O3—H4⋯O5 hydrogen bonds, which form centrosymmetric loops with graph set 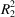 (8) (Etter, 1990 ▸; Bernstein *et al.*, 1995 ▸; Shields *et al.*, 2000 ▸). These hydrogen bonds link the mol­ecules into chains propagating along [011] (Table 1 ▸; Fig. 5 ▸). There is an intra­molecular O1—H2⋯O5 hydrogen bond between the hydroxyl group and the carbonyl group of the carboxyl acid. A C—H⋯O hydrogen bond also contributes to the lattice energy. The *Mercury* aromatics analyser indicates one strong inter­action with a centroid–centroid distance of 4.26 Å, and a moderate one at 5.59 Å.

**Figure 6:**
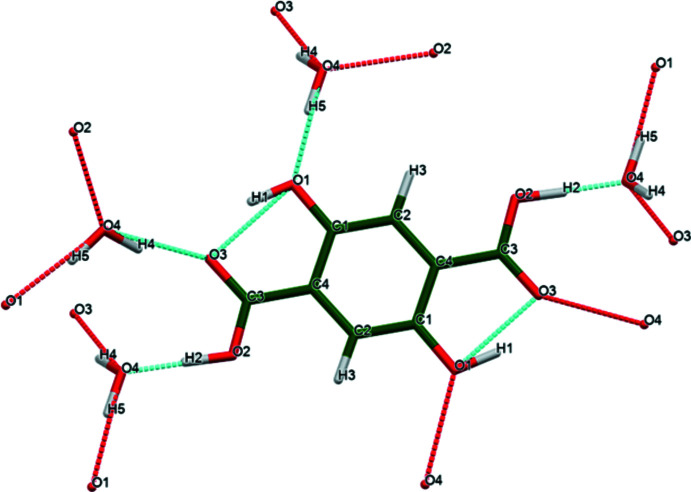
The hydrogen bonds in the structure of 2–5-di­hydroxy­terephthalic acid dihydrate DUSJUX. Image generated using *Mercury* (Macrae *et al.*, 2020 ▸).

The hydrogen bonding in the dihydrate DUSJUX (Cheng *et al.*, 2010 ▸) is very different (Table 2 ▸; Fig. 6 ▸). Although the intra­molecular hy­droxy–carb­oxy­lic acid O—H⋯O hydrogen bond is present, the water mol­ecule acts as an acceptor to the carb­oxy­lic acid and a donor to two other oxygen atoms. The carb­oxy­lic acid groups do not inter­act with each other directly.

The *CRYSTAL17* (Dovesi *et al.*, 2018 ▸) calculations suggest that DUSJUX is 28.5 kcal mol^−1^ lower in energy than the sum of anhdyrous 2,5-di­hydroxy­terephthalic acid and two water mol­ecules. The corresponding *VASP* (Kresse & Furthmüller, 1996 ▸) calculations indicate that DUSJUX is 114.0 kcal mol^−1^ more stable. As chemists, we would like to attribute the ‘extra’ energy to the formation of additional hydrogen bonds. Rammohan & Kaduk (2018 ▸) developed (for citrates using earlier versions of *CRYSTAL*) a correlation between the energy of an O—H⋯O hydrogen bond and the Mulliken overlap population between the H and the O acceptor: *E* (kcal mol^−1^) = 54.7(overlap)^1/2^. Using this correlation to estimate the energies of the individual hydrogen bonds, we calculate that DUSJUX is 59.6 kcal mol^−1^ lower in energy than the sum of the anhydrous mol­ecule and two water mol­ecules – a value between the two DFT calculations. While the disagreements indicate that the absolute energy calculated for a hydrogen bond may be more uncertain than we would like, the Mulliken overlap population is certainly a guide to whether a hydrogen bond is stronger or weaker than another, and to whether a (geometrically possible) hydrogen bond is real or not.

## 4. Database survey

A connectivity search in the Cambridge Structural Database [CSD version 5.43 June 2022 (Groom *et al.*, 2016 ▸); *ConQuest* 2022.2.0 (Bruno *et al.*, 2002 ▸)] of a 2,5-di­hydroxy­terephthalate fragment with the elements C, H, and O only yielded the structure of the dihydrate (Cheng *et al.*, 2010 ▸; DUSJUX), as well as two esters. The dihydrate was also obtained accidentally during the synthesis of metal–organic coordination polymers. Removing the chemistry limitation yielded 249 entries, many of which are metal–organic frameworks. A search of the powder pattern against the Powder Diffraction File (Gates-Rector & Blanton, 2019 ▸) yielded no hits. Not even the usual accidental matches were obtained; this pattern evidently occupies a very different portion of ‘diffraction space’.

## 5. Synthesis and crystallization

Cobalt(II) chloride hexa­hydrate (1.78 g, 7.50 mmol) and 2,5-di­hydroxy­terephthalic acid (1.00 g, 5.05 mmol) were crushed together with mortar and pestle and added to a 10 ml round-bottom flask. The flask was connected to a Schlenk line and placed in a glass bowl of sand on top of a hot plate. The hot plate was heated to 443 K for approximately 18 h and the round-bottom flask was under vacuum. After being removed from the heat and allowed to cool, the remaining solid was transferred to a Pyrex container with aceto­nitrile (50 ml) and placed in a vacuum oven at 343 K for 24 h. After removal from the oven, the solution was deca­nted and replaced with aceto­nitrile (50 ml). This wash procedure was done a total of three times. The remaining solid was then added to 100 ml of methanol at 343 K for 24 h and deca­nted, this wash was done two times. The remaining solid was then added to a vacuum oven at 423 K for 24 h. The remaining solid was then added to a scintillation vial wrapped with Parafilm for storage.

## 6. Refinement

Crystal data, data collection and structure refinement details are summarized in Table 3 ▸. A portion of the sample was blended with 11.51% < 1 micron diamond powder (Alfa Aesar) inter­nal standard in a mortar and pestle until the color was uniform. The X-ray powder diffraction pattern was measured from a 0.7 mm diameter static capillary specimen on a PANalytical Empyrean diffractometer using Mo *K*α radiation. The pattern was measured from 1.0–50.0° 2θ in 0.0083560° steps, counting for 4 sec/step.

After correcting the peak positions using the known diamond peak positions, the pattern was indexed using *JADE Pro* (MDI, 2022 ▸) on a primitive triclinic cell with *a* = 4.26420, *b* = 5.58601, *c* = 8.17902 Å, *α* = 93.53, *β* = 12.13, *γ* = 96.78° and *V* = 188 Å^3^. Since the volume corresponded to one mol­ecule of 2,5-di­hydroxy­terephthalic acid, the space group was assumed to be *P* 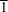 , with half a mol­ecule in the asymmetric unit. A reduced cell search of the CSD yielded no hits. Preliminary indexing attempts using the default peak list from a pattern collected using Cu radiation were unsuccessful (monoclinic cells with no reasonable structures), until closer examination of the pattern revealed that the peak at 21.6° (9.7° Mo) was actually a doublet, and that there was an additional peak at 22.0° (9.9° Mo). Including these two additional peaks yielded the triclinic cell.

The 2,5-di­hydroxy­terephthalic acid mol­ecule was extracted from the DUSJUX structure using *Materials Studio* (Dassault Systèmes, 2021 ▸), and saved as a .mol2 file. The crystal structure was solved using Monte Carlo simulated annealing techniques as implemented in *EXPO2014* (Altomare *et al.*, 2013 ▸), using a whole mol­ecule as the fragment. Since the mol­ecule occupies a center of symmetry, the two halves overlapped partially. The overlapping atoms were averaged manually using *Materials Studio* to obtain the asymmetric unit.

**Figure 7:**
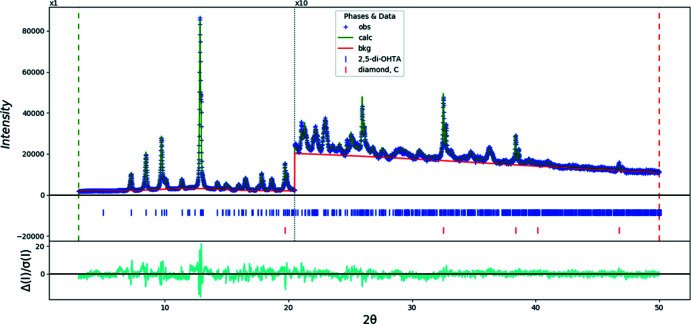
The Rietveld plot for the refinement of anhydrous 2,5-di­hydroxy­terephthalic acid. The blue crosses represent the observed data points, and the green line is the calculated pattern. The cyan curve is the normalized error plot, and the red line is the background curve. The row of tick marks indicates the calculated reflection positions. The vertical scale has been multiplied by a factor of 10× for 2θ > 20.5°. The row of red tick marks indicate the positions of the diamond internal standard peaks.

Rietveld refinement was carried out using *GSAS-II* (Toby & Von Dreele, 2013 ▸). All non-H bond distances and angles were subjected to restraints, based on a *Mercury* Mogul geometry check (Sykes *et al.*, 2011 ▸; Bruno *et al.*, 2004 ▸). A planar restraint was applied to the benzene ring. The Mogul average and standard deviation for each qu­antity were used as the restraint parameters. The restraints contributed 1.9% to the final χ^2^. The hydrogen atoms were included in calculated positions, which were recalculated during the refinement using *Materials Studio* (Dassault Systèmes, 2021 ▸). The *U_iso_* of the heavy atoms were grouped by chemical similarity. The *U*_iso_ for the H atoms were fixed at 1.3× the *U*_iso_ of the heavy atoms to which they are attached. The peak profiles were described using the generalized microstrain model. The background was modeled using a four-term shifted Chebyshev polynomial, along with a peak at 12.05° to model the scattering from the glass capillary and any amorphous component. The final refinements yielded the residuals reported in Table 1 ▸. The largest errors in the difference plot (Fig. 7 ▸) are small, and are in the shapes of the peaks.

The crystal structure (as well as that of DUSJUX and an isolated water mol­ecule) was optimized using *VASP* (Kresse & Furthmüller, 1996 ▸) (fixed experimental unit cells) through the *MedeA* graphical inter­face (Materials Design, 2016 ▸). The calculations were carried out on 16 2.4 GHz processors (each with 4 Gb RAM) of a 64-processor HP Proliant DL580 Generation 7 Linux cluster at North Central College. The calculations used the GGA-PBE functional, a plane wave cutoff energy of 400.0 eV, and a *k*-point spacing of 0.5 Å^−1^ leading to a 4 × 3 × 2 mesh. The structures were also optimized (fixed experimental cells) and population analyses were carried out using *CRYSTAL17* (Dovesi *et al.*, 2018 ▸). The basis sets for the H, C, N, and O atoms in the calculations were those of Gatti *et al.* (1994 ▸). The calculations were run on a 3.5 GHz PC using 8 *k*-points and the B3LYP functional.

## Supplementary Material

Crystal structure: contains datablock(s) global, I_DFT, DUSJUX_DFT, vege083_overall, I, Ia, vege083_pwd_0. DOI: 10.1107/S2056989022009409/hb8038sup1.cif

Supporting information file. DOI: 10.1107/S2056989022009409/hb8038Isup2.cml

CCDC references: 2209514, 2209515, 2209516, 2209517

Additional supporting information:  crystallographic information; 3D view; checkCIF report

## Figures and Tables

**Table 1:** Hydrogen-bond geometry (Å, °) for (I)

*D*—H⋯*A*	*D*—H	H⋯*A*	*D*⋯*A*	*D*—H⋯*A*
O3—H4⋯O5^i^	1.00	1.69	2.689	174
O1—H2⋯O5	0.99	1.68	2.567	147

Symmetry code: (i) 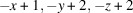 .

**Table 2:** Hydrogen-bond geometry (Å, °) for DUSJUX[Chem scheme1]

*D*—H⋯*A*	*D*—H	H⋯*A*	*D*⋯*A*	*D*—H⋯*A*
O2—H2⋯O4	1.07	1.43	2.500	178
O1—H1⋯O3^i^	1.01	1.64	2.562	149
O4—H4⋯O3^ii^	0.99	1.78	2.736	161
O4—H5⋯O1^iii^	0.99	1.82	2.794	169

Symmetry codes: (i) 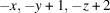 ; (ii) 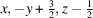 ; (iii) 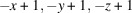 .

**Table 3:** Experimental details

	(I)	DUSJUX (DFT)
Crystal data		
Chemical formula	C_8_H_6_O_6_	C_8_H_6_O_6_·2(H_2_O)
*M*_r_	198.08	--
Crystal system, space group	Triclinic, *P* 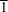	Monoclinic, *P*2_1_/*c*
Temperature (K)	302	--
*a*, *b*, *c* (Å)	4.2947 (5), 5.6089 (5), 8.2331 (19)	5.18830, 17.54500, 5.49900
α, β, γ (°)	93.612 (4), 102.219 (4), 96.7621 (14)	90, 103.03, 90
*V* (Å^3^)	191.69 (1)	487.68
*Z*	1	2
Radiation type	Mo *K*α_1,2_, λ = 0.70932, 0.71361 Å	--
Specimen shape, size (mm)	Cylinder, 12 × 0.7	--

Data collection		
Diffractometer	PANalytical Empyrean	
Specimen mounting	Glass capillary	
Data collection mode	Transmission	
Data collection method	Step	
θ values (°)	2θ_min_ = 1.002 2θ_max_ = 49.991 2θ_step_ = 0.008	

Refinement		
*R* factors and goodness of fit	*R*_p_ = 0.034, *R*_wp_ = 0.042, *R*_exp_ = 0.019, χ^2^ = 5.148	
No. of parameters	53	
No. of restraints	18	
(Δ/σ)_max_	2.635	

The same symmetry and lattice parameters were used for the DFT calculations as for the powder diffraction study for (I). Computer program: *GSAS-II* (Toby & Von Dreele, 2013 ▸).

## supplementary crystallographic information

### 2,5-Dihydroxybenzene-1,4-dicarboxylic acid (I) Crystal data

**Table:**

C_8_H_6_O_6_	β = 102.219 (4)°
*M**_r_* = 198.08	γ = 96.7621 (14)°
Triclinic, *P*1	*V* = 191.69 (1) Å^3^
Hall symbol: -P 1	*Z* = 1
*a* = 4.2947 (5) Å	*D*_x_ = 1.716 Mg m^−^^3^
*b* = 5.6089 (5) Å	*T* = 302 K
*c* = 8.2331 (19) Å	cylinder, 12 × 0.7 mm
α = 93.612 (4)°	

### 2,5-Dihydroxybenzene-1,4-dicarboxylic acid (I) Data collection

**Table:**

PANalytical Empyrean diffractometer	Data collection mode: transmission
Specimen mounting: glass capillary	Scan method: step

### 2,5-Dihydroxybenzene-1,4-dicarboxylic acid (I) Refinement

**Table:**

18 restraints	Preferred orientation correction: Simple spherical harmonic correction Order = 4 Coefficients: 0:0:C(2,-2) = 0.246(11); 0:0:C(2,-1) = -0.018(11); 0:0:C(2,0) = -0.313(16); 0:0:C(2,1) = 0.217(13); 0:0:C(2,2) = -0.192(9); 0:0:C(4,-4) = -0.146(17); 0:0:C(4,-3) = 0.073(19); 0:0:C(4,-2) = -0.052(16); 0:0:C(4,-1) = 0.083(18); 0:0:C(4,0) = -0.058(17); 0:0:C(4,1) = -0.006(18); 0:0:C(4,2) = -0.196(23); 0:0:C(4,3) = 0.071(16); 0:0:C(4,4) = 0.108(25)

### 2,5-Dihydroxybenzene-1,4-dicarboxylic acid (I) Fractional atomic coordinates and isotropic or equivalent isotropic displacement parameters (Å^2^)

**Table:**

	*x*	*y*	*z*	*U*_iso_*/*U*_eq_	
C10	0.492 (2)	0.6406 (16)	0.6392 (11)	0.0323 (10)*	
C6	0.6818 (18)	0.4506 (18)	0.6571 (9)	0.0323 (10)*	
C7	0.6946 (18)	0.3106 (14)	0.5107 (12)	0.0323 (10)*	
C9	0.477 (2)	0.7892 (16)	0.7918 (9)	0.0553 (15)*	
O1	0.8599 (12)	0.4082 (9)	0.8120 (6)	0.0323 (10)*	
O3	0.2791 (12)	0.9640 (12)	0.7718 (7)	0.0553 (15)*	
O5	0.6452 (16)	0.7744 (11)	0.9376 (9)	0.0553 (15)*	
H8	0.84185	0.17135	0.52928	0.0420 (14)*	
H2	0.83782	0.54288	0.88998	0.0420 (14)*	
H4	0.30280	1.07255	0.87663	0.0719 (19)*	

### 2,5-Dihydroxybenzene-1,4-dicarboxylic acid (I) Geometric parameters (Å, º)

**Table:**

C10—C6	1.411 (5)	C9—O3	1.365 (5)
C10—C7^i^	1.384 (5)	C9—O5	1.277 (5)
C10—C9	1.482 (6)	O1—C6	1.391 (5)
C6—C10	1.411 (5)	O1—H2	0.987 (5)
C6—C7	1.412 (6)	O3—C9	1.365 (5)
C6—O1	1.391 (5)	O3—H4	1.004 (6)
C7—C10^i^	1.384 (5)	O5—C9	1.277 (5)
C7—C6	1.412 (6)	H8—C7	1.060 (8)
C7—H8	1.060 (8)	H2—O1	0.987 (5)
C9—C10	1.482 (6)	H4—O3	1.004 (6)
			
C6—C10—C7^i^	124.3 (7)	C10^i^—C7—H8	126.7 (10)
C6—C10—C9	118.1 (9)	C6—C7—H8	115.1 (10)
C7^i^—C10—C9	117.5 (9)	C10—C9—O3	116.8 (7)
C10—C6—C7	117.4 (6)	C10—C9—O5	125.0 (9)
C10—C6—O1	121.6 (9)	O3—C9—O5	118.1 (7)
C7—C6—O1	121.0 (10)	C6—O1—H2	105.5 (6)
C10^i^—C7—C6	118.1 (7)	C9—O3—H4	112.7 (5)

Symmetry code: (i) −*x*+1, −*y*+1, −*z*+1.

### (Ia) Crystal data

**Table:**

C	*V* = 46.12 (1) Å^3^
*M**_r_* = 12.01	*Z* = 8
Cubic, *F**d*3*m*	*D*_x_ = 3.459 Mg m^−^^3^
Hall symbol: -F 4vw 2vw	*T* = 302 K
*a* = 3.58625 (11) Å	

### (Ia) Refinement

**Table:**

Preferred orientation correction: March-Dollase correction coef. = 1.000 axis = [0, 0, 1]	

### (Ia) Fractional atomic coordinates and isotropic or equivalent isotropic displacement parameters (Å^2^)

**Table:**

	*x*	*y*	*z*	*U*_iso_*/*U*_eq_	
C1	0.12500	0.12500	0.12500	0.0159*	

### (Ia) Geometric parameters (Å, º)

**Table:**

C1—C1^i^	1.5529	C1—C1^iii^	1.5529
C1—C1^ii^	1.5529	C1—C1^iv^	1.5529
			
C1^i^—C1—C1^ii^	109.471	C1^i^—C1—C1^iv^	109.471
C1^i^—C1—C1^iii^	109.471	C1^ii^—C1—C1^iv^	109.471
C1^ii^—C1—C1^iii^	109.471	C1^iii^—C1—C1^iv^	109.471

Symmetry codes: (i) *x*+1/4, *y*+1/4, −*z*; (ii) −*z*, *x*+1/4, *y*+1/4; (iii) *y*+1/4, −*z*, *x*+1/4; (iv) −*x*, −*y*, −*z*.

### (I_DFT) Crystal data

**Table:**

C_8_H_6_O_6_	*c* = 8.1976 Å
*M**_r_* = 198.08	α = 93.6590°
Triclinic, *P*1	β = 102.1730°
*a* = 4.2647 Å	γ = 96.7840°
*b* = 5.5912 Å	*Z* = 1

### (I_DFT) Data collection

**Table:**

*h* = →	*l* = →
*k* = →	

### (I_DFT) Fractional atomic coordinates and isotropic or equivalent isotropic displacement parameters (Å^2^)

**Table:**

	*x*	*y*	*z*	*U*_iso_*/*U*_eq_	
C10	0.50725	0.65165	0.64068	0.06414*	
C6	0.68695	0.46350	0.65571	0.06414*	
C7	0.69097	0.31393	0.51546	0.06414*	
C9	0.48403	0.80691	0.79069	0.01062*	
O1	0.87656	0.42423	0.80458	0.01062*	
O3	0.28942	0.97462	0.76830	0.01062*	
O5	0.65017	0.78063	0.93109	0.01062*	
H8	0.84185	0.17135	0.52928	0.08339*	
H2	0.83782	0.54288	0.88999	0.01381*	
H4	0.30280	1.07255	0.87663	0.01381*	

### (I_DFT) Bond lengths (Å)

**Table:**

C10—C6	1.370	C7—H8	1.079
C10—C7^i^	1.415	C9—O3	1.320
C10—C9	1.487	C9—O5	1.245
C6—C7	1.382	O1—H2	0.986
C6—O1	1.361	O3—H4	1.000
C7—C10^i^	1.415	H4—O3	1.000

Symmetry code: (i) −*x*+1, −*y*+1, −*z*+1.

### (I_DFT) Hydrogen-bond geometry (Å, º)

**Table:**

*D*—H···*A*	*D*—H	H···*A*	*D*···*A*	*D*—H···*A*
O3—H4···O5^ii^	1.00	1.69	2.689	174
O1—H2···O5	0.99	1.68	2.567	147

Symmetry code: (ii) −*x*+1, −*y*+2, −*z*+2.

### (DUSJUX_DFT) Crystal data

**Table:**

C_8_H_6_O_6_·2(H_2_O)	*c* = 5.49900 Å
Monoclinic, *P*2_1_/*c*	β = 103.03°
*a* = 5.18830 Å	*V* = 487.68 Å^3^
*b* = 17.54500 Å	*Z* = 2

### (DUSJUX_DFT) Data collection

**Table:**

*h* = →	*l* = →
*k* = →	

### (DUSJUX_DFT) Fractional atomic coordinates and isotropic or equivalent isotropic displacement parameters (Å^2^)

**Table:**

	*x*	*y*	*z*	*B*_iso_*/*B*_eq_	
O1	0.02829	0.35100	0.84164		
H1	−0.08229	0.31915	0.93268		
O2	0.41842	0.59430	0.63866		
H2	0.51266	0.64044	0.56603		
O3	0.28123	0.68492	0.87357		
C1	0.01145	0.42401	0.92007		
C2	0.14692	0.48106	0.82449		
H3	0.26253	0.46707	0.68792		
C3	0.28585	0.61663	0.80175		
C4	0.13841	0.55682	0.90233		
O4	0.64658	0.70011	0.46632		
H4	0.52537	0.74263	0.39890		
H5	0.75145	0.68680	0.34282		

### (DUSJUX_DFT) Hydrogen-bond geometry (Å, º)

**Table:**

*D*—H···*A*	*D*—H	H···*A*	*D*···*A*	*D*—H···*A*
O2—H2···O4	1.07	1.43	2.500	178
O1—H1···O3^i^	1.01	1.64	2.562	149
O4—H4···O3^ii^	0.99	1.78	2.736	161
O4—H5···O1^iii^	0.99	1.82	2.794	169

Symmetry codes: (i) −*x*, −*y*+1, −*z*+2; (ii) *x*, −*y*+3/2, *z*−1/2; (iii) −*x*+1, −*y*+1, −*z*+1.
